# Nonadherent culture method promotes MSC-mediated vascularization in myocardial infarction via miR-519d/VEGFA pathway

**DOI:** 10.1186/s13287-020-01780-x

**Published:** 2020-07-02

**Authors:** Baoping Deng, Xianlan Zhang, Yi Liang, Haiming Jiang, Weizhao Huang, Yinmeng Wu, Weiping Deng

**Affiliations:** 1 Department of Cardiothoracic Surgery, Zhongshan People’s Hospital, 2 Sunwen East Road, Zhongshan, Guangdong 528403 People’s Republic of China; 2 Department of Vascular Surgery, Affiliated Hospital of Guilin Medical University, Guilin, 541001 People’s Republic of China; 3 Department of Gastroenterology, Taihe Hospital, Hubei University of Medicine, 32 Ren Min South Road, Shiyan, 442000 Hubei People’s Republic of China

**Keywords:** Mesenchymal stem cells, Myocardial infarction, VEGFA, Angiogenesis, miR-519d, Nonadherent culture

## Abstract

**Background:**

Mesenchymal stem cells (MSCs) can provide therapeutic benefits for myocardial infarction (MI) recovery; however, the molecular mechanism by which MSCs improve the heart function is unclear.

**Methods:**

Microarray analysis was performed to examine the expression profiling of human MSCs (hMSCs) grown as adherent cultures (AC-hMSCs) or nonadherent cultures on ultra-low-adherent plates (nonAC-hMSCs). Real-time quantitative polymerase chain reaction (RT-qPCR), western blotting, and enzyme-linked immunosorbent assays (ELISA) were used to assess VEGFA expression and secretion in the AC-hMSCs and nonAC-hMSCs. The paracrine effect of VEGFA-overexpressing AC-MSCs (AC-VEGFA-hMSCs) or VEGFA-knockdown nonAC-hMSCs (nonAC-shVEGFA-hMSCs) on the angiogenic ability of human umbilical vein endothelial cells (HUVECs) was evaluated using tube formation assay. AC-VEGFA-hMSCs or nonAC-shVEGFA-hMSCs were transplanted into myocardial infarction rats to investigate the therapeutic effect of AC-VEGFA-hMSCs or nonAC-shVEGFA-hMSCs. Luciferase reporter assay was used to confirm the association of VEGFA with miR-519d.

**Results:**

Microarray analysis revealed that VEGFA is downregulated in AC-hMSCs compared to nonAC-hMSCs. Functional assays revealed that high levels of VEGFA produced from AC-VEGFA-hMSCs increased the tube formation capacity of HUVECs in vitro, improved angiogenesis and cardiac performance, and reduced infarct size in a rat MI model. Low levels of VEGFA secretion from nonAC-shVEGFA-hMSCs had the opposite effects. Mechanistically, we found that miR-519d directly targets VEGFA. High levels of VEGFA secreted from VEGFA-overexpressing nonAC-hMSCs abolished the repressive effect of miR-519d on HUVEC angiogenesis.

**Conclusion:**

Our findings indicate that nonadherent culture-induced secretion of VEGFA plays an important role in MSCs via the miR-519d/VEGFA pathway and may provide a novel therapeutic strategy for MI treatment.

## Introduction

Myocardial infarction (MI) is a major cause of mortality and disability in the world [1]. Pharmacologic intervention and conventional revascularization techniques, such as coronary artery bypass grafting, balloon angioplasty, and stenting, can restore blood flow and maintain myocardial viability and function; however, these treatment methods cannot salvage the dying myocardium or repair cardiac function [2]. In recent years, stem cell therapy has become a promising approach to treat MI, as it has the potential to restore damaged myocardium [3, 4]. Mesenchymal stem cells (MSCs) exist in the bone marrow, umbilical cord blood, adipose, and many other tissues [5] and have multilineage differentiation potential, self-renewal capacity, and immunomodulatory properties [6], while posing a low immunorejection risk [7]. Thus, MSCs have been commonly used in experimental research and clinical trials for treating MI. Often for this application, a large amount of MSCs must be isolated from the same tissues and expanded in plastic adherent culturing containers [7, 8]. It was recently reported that in vitro expansion in conventional monolayer cultures can alter the phenotype of MSCs, which may cause cell trapping within the lung and a low rate of delivery to target organs [9–12]. Qian et al. [13] showed that primary MSCs lacked CD44, whereas culture-expanded MSCs acquired CD44 expression. Compared to primary MSCs, cultured MSCs with high levels of CD44 displayed decreased targeting to the bone marrow [13]. Our previous research revealed that stem cell antigen 1 (Sca-1) is expressed at higher levels in adherent cultured mouse MSCs (AC-mMSCs) compared to mMSCs in nonadherent cultures maintained in ultra-low-adherent plates (nonAC-mMSCs) [14]. Sca-1^+^ mMSCs play a crucial role in improving cardiac function in MI [14, 15]. Compared with nonadherent cultured human MSCs (nonAC-hMSCs), adherent cultured hMSCs (AC-hMSCs) with high protein expression of caspase-3, caspase-7, and caspase-9 had a marked decrease in cell apoptosis [16]. Therefore, it is important to investigate the changes in MSC phenotype that occur during in vitro cultivation.

MSC therapy with angiogenic factors holds a great promise for ischemic disease treatment due to neovascularization [17, 18]. Vascular endothelial growth factor A (VEGFA) is a major driver of angiogenesis and vasculogenesis [19]. VEGFA could regulate angiogenesis and myogenesis in cardiac repair [20, 21]. A growing body of research demonstrated that VEGFA promotes MSC viability in the infarcted hearts via decreasing cellular stress and enhancing cell survival factors [22, 23]. Therefore, it is anticipated that VEGFA-gene-modified MSCs may provide a potentially valuable approach for MI treatment due to increase survival and angiogenic capacity.

In this study, we identified that VEGFA expression is significantly higher in nonAC-hMSCs than in AC-hMSCs, as detected by microarray analysis. The increased levels of VEGFA secreted from VEGFA-overexpressing AC-hMSCs (AC-VEGFA-hMSCs) facilitated tube formation of human umbilical vein endothelial cells (HUVECs), while the decreased levels of VEGFA secreted by nonAC-hMSCs with VEGFA knockdown (nonAC-shVEGFA-hMSCs) led to the opposite effect. In a rat MI model, the increased levels of VEGFA released from AC-VEGFA-hMSCs promoted angiogenesis, decreased infarct size, and improved myocardial function, while the decreased levels of VEGFA secreted by nonAC-shVEGFA-hMSCs reduced angiogenesis, increased infarct size, and impaired cardiac remodeling. Further studies on the mechanism underlying these changes indicated that VEGFA is a direct target of miR-519d. High levels of VEGFA produced by VEGFA-overexpressing nonAC-hMSCs reversed the inhibitory effect of miR-519d on the tube formation capability of HUVECs.

## Materials and methods

### Isolation and expansion of hMSCs and in vitro nonadherent culture conditions

hMSCs were isolated from the bone marrow of adult donors (ages 18–25) who underwent orthopedic surgery as described previously [16]. hMSCs were seeded in adherent culture plates (AC-hMSCs) or ultra-low-adherent tissue culture plates (nonAC-hMSCs; Corning®, Corning, NY) and grown in Dulbecco’s modified Eagle’s medium (DMEM; GIBCO, Grand Island, NY) for 24 h or 72 h. All cells were incubated in a humidified incubator with 5% CO_2_ at 37 °C. This study was approved by the Ethics Committee of Affiliated Hospital of Guilin Medical University (Guilin, China), and informed consent was obtained from all participants.

### Flow cytometry

Cells were harvested, washed, and resuspended. The antibodies included anti-CD11b (BD Biosciences, Franklin Lakes, NJ, USA), anti-CD14 (BD Biosciences), anti-CD34 (BD Biosciences), anti-CD45 (BD Biosciences), anti-CD73 (BD Biosciences), anti-CD90 (BD Biosciences), and anti-CD105 (BD Biosciences) were added to the cells at 4 °C for 1 h. Then, cells were analyzed on a BD FACSCalibur (BD Biosciences) and data was analyzed by BD FACSComp software (BD Biosciences). An isotype control immunoglobulin (BD Biosciences) was used as control.

### mRNA microarray analysis

AC-hMSCs and nonAC-hMSCs grown for 24 h and 72 h were sent to the Shanghai Bohao Biotechnological Co., Ltd. (Shanghai, China), for mRNA microarray profiling. Microarray data were analyzed using GeneSping software (Agilent Technologies, Santa Clara, CA). The log_2_-fold change was determined by calculating gene expression in AC-hMSCs and nonAC-hMSCs. Differentially expressed genes were identified using a *t* test and Benjamini-Hochberg correction (corrected *P* < 0.05 and fold change > 2).

### Plasmid construction, oligonucleotide, and transfection

The full-length VEGFA cDNA was PCR-amplified and inserted into a pcDNA3.1 plasmid. miR-519d mimics, miR-519d inhibitors, and the appropriate controls were purchased from Ribobio (Guangzhou, China). Transient transfection was performed using Lipofectamine 2000 (Invitrogen, Carlsbad, CA) according to the manufacturer’s instructions.

### Lentiviruses and infection

VEGFA, shVEGFA, and the corresponding control lentiviruses also carrying the GFP coding sequence were obtained from GeneChem Co., Ltd. (Shanghai, China). AC-hMSCs and nonAC-hMSCs were transduced with VEGFA or shVEGFA lentiviruses (AC-VEGFA-hMSCs or nonAC-shVEGFA-hMSCs), respectively. An inverted fluorescence microscope was used to detect GFP expression.

### Real-time quantitative polymerase chain reaction (RT-qPCR)

Total RNA was extracted from cultured cells using Trizol reagent (Invitrogen). A microRNA Reverse Transcription Kit (Promega, Madison, WI, USA) or a PrimeScript RT-PCR kit (Takara, Dalian, China) was used to synthesize cDNA. RT-qPCR was carried out using SYBR Premix Ex Taq II (TaKaRa). Relative quantification of gene expression was determined using the 2^-△△Ct^ method [24]. The following primers were used: VEGFA primers: forward 5′-GCAGAATCATCACGAAGTGGTG-3′, reverse 5′-TCTCGATTGGATGGCAGTAGCT-3′; β-actin primers: forward 5′-CTCCATCCTGGCCTCGCTGT-3′, reverse 5′-ACTAAGTCATAGTCCGCCTAGA-3′; miR-519d RT primers: 5′-GTCGTATCCAGTGCAGGGTCCGAGGTATTCGCACTGGATACGACCACTCT-3′, PCR primers: forward 5′-GCCAAAGTGCCTCCCTTT-3′, reverse 5′-GTGCAGGGTCCGAGGT-3′; U6 primers: forward 5′-CTCGCTTCGGCAGCACA-3′, reverse 5′-AACGCTTCACGAATTTGCGT-3′.

### Enzyme-linked immunosorbent assays (ELISA)

Conditioned medium (CM) was obtained from different MSC groups and centrifuged for further experiments. Rat heart tissues were homogenized in 20 mL of PBS, subjected to two freeze-thaw cycles, and centrifuged for further experiments. The secreted levels of VEGFA in the CM and rat heart tissues were examined using a VEGFA ELISA Kit (R&D System, Los Angeles, CA) according to the manufacturer’s protocol, and the absorbance at 450 nm was measured using a microplate reader. Each sample was assayed three times.

### Tube formation assay

HUVECs were obtained from the Jennio Biotech (Guangzhou, China). HUVECs (2 × 10^4^ cells) were suspended in CM obtained from different hMSC groups, added to the 96-well plates coated with Matrigel (BD Biosciences, Franklin Lakes, NJ) at a density of 2 × 10^4^ cells/well, and incubated at 37 °C for 12 h. Images of tubular structures were captured and analyzed using Image-Pro Plus 6.0 software.

### Rat MI model and hMSC transplantation

Male Sprague-Dawley rats weighing 250–350 g were subjected to MI using left anterior descending coronary artery (LADCA) ligation. After anesthetized with 10% chloral hydrate (250 mg/kg weight), the rats were intubated with polyethylene-16 tube and connected to the rodent ventilator (Harvard Apparatus, Holliston, MA). The chest was opened between the third and fourth ribs, and the pericardium was split to expose the left ventricle (LV), aorta, and left atrium. The LADCA was ligated using a 7-0 polypropylene suture 2 mm below the edge of the left atrium. Forty-eight rats were randomly divided into six treatment groups: rats that received AC-VEGFA-hMSCs (*n* = 8), nonAC-shVEGFA-hMSCs (*n* = 8), AC-hMSCs (*n* = 8), nonAC-hMSCs (*n* = 8), saline (MI group, *n* = 8), or saline (a sham operation, *n* = 8) in the infarct border region immediately after LAD ligation. The chest and skin were then closed. All experiments were approved by the Ethics Committee of the Affiliated Hospital of Guilin Medical University.

### Cardiac function assessment

Four weeks after LADCA ligation, an echocardiography was performed using a Vevo 2100 system (Visualsonics, Toronto, ON, Canada) with a 21-MHz transducer. Rats were anesthetized with 10% chloral hydrate (250 mg/kg weight) for the exam. LV end-diastolic and end-systolic dimensions (LVEDD, LVESD) from the parasternal short-axis view at the papillary muscle were measured using M-mode tracing [25]. LV ejection fraction (LVEF) was calculated using the following formula: LVEF (%) = (LVEDD^3^ - LVESD^3^)/LVEDD^3^ × 100. LV fractional shortening (LVFS) was calculated as LVFS (%) = (LVEDD - LVESD)/LVEDD × 100. Measurements were done in triplicate.

### Measurement of LV infarct size

The heart tissues were fixed in 10% formalin, embedded in paraffin, and cut into 5 μm slices. Sections were then stained with Masson’s trichrome according to the manufacturer’s instructions and examined using an optical microscope (Olympus, Tokyo, Japan), and images were acquired using a Retiga CCD camera. Image-Pro Plus 6.0 software was used to measure the infarct area and total LV area of each image. Infarct size was expressed as a percentage of the total LV area.

### Immunohistochemical staining

Sections were dewaxed with xylene and rehydrated with graded ethanol. Subsequently, sections were pretreated with 0.01 M citrate buffer (pH 6.0) in a microwave oven at 95 °C for 20 min and incubated with 3% H_2_O_2_ for 15 min at room temperature. The sections were blocked with 10% sheep serum for 30 min and then incubated with anti-Von Willebrand Factor (vWF, Abcam, Cambridge, MA) primary antibody overnight at 4 °C. After washes with PBS, sections were incubated with secondary antibody for 30 min at room temperature and stained with diaminobenzidine. After counterstaining with hematoxylin, sections were analyzed using an optical microscope (Olympus) and photographed using a digital camera.

### Luciferase reporter assay

The VEGFA 3′ UTR, which contains a putative miR-519d binding site, was PCR-amplified and inserted into the psiCHECK-2 luciferase reporter plasmid (VEGFA-3′ UTR-WT; Promega). Site-directed mutagenesis (Stratagene, La Jolla, CA) was used to construct the mutant VEGFA 3′ UTR (Mut), which was then cloned into the psiCHECK-2 luciferase reporter plasmid (VEGFA-3′ UTR-Mut). VEGFA-3′ UTR-WT or VEGFA-3′ UTR-Mut vectors were transfected into HEK293T cells. Luciferase activity was analyzed 48 h after transfection using a dual-luciferase reporter assay kit (Promega). Data are represented as the Renilla/firefly luciferase ratio.

### Western blotting

Tissues and cells were lysed with RIPA lysis buffer (Beyotime Biotechnology, Shanghai, China) containing protease inhibitor. Protein extracts were separated by SDS-PAGE and transferred onto a PVDF membrane (Millipore, Billerica, MA). The membrane was blocked with 5% non-fat milk and incubated with anti-VEGFA primary antibody (Abcam) at 4 °C overnight, followed by incubation with HRP-conjugated secondary antibody at room temperature. Protein signals were visualized using an ECL detection kit (Millipore).

### Statistical analysis

The data analysis was performed using SPSS 20.0 software. The differences between two groups were evaluated using student’s *t* tests. Values are expressed as the mean ± standard deviation. *P* values < 0.05 were considered statistically significant.

## Results

### Increased VEGFA expression and production are observed in nonAC-hMSCs

**Fig. 1:**
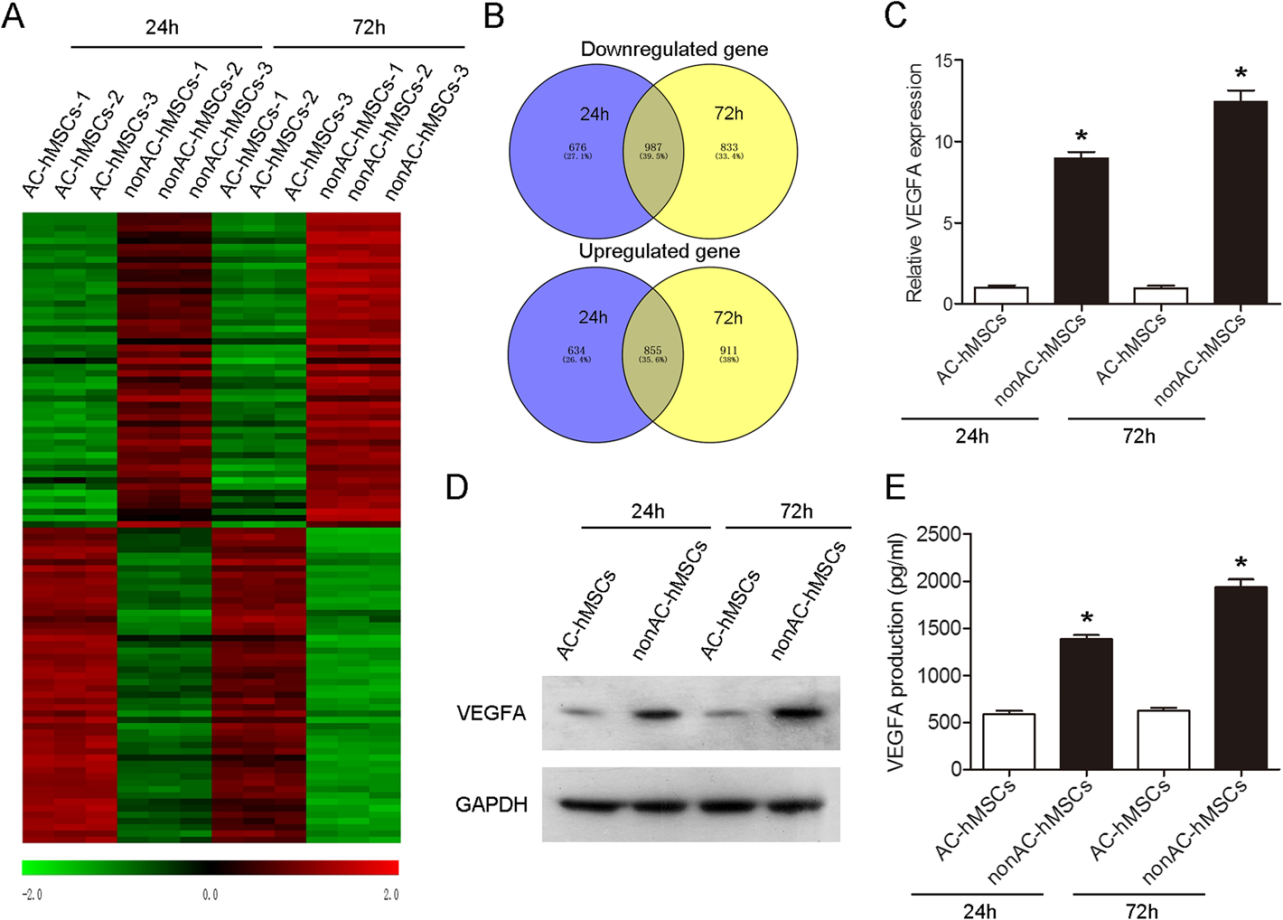
Increased VEGFA expression is observed in the nonAC-hMSCs at two different time points. **a** The 50 most downregulated and 50 most upregulated mRNAs in the AC-hMSCs and nonAC-hMSCs at 24 h and 72 h. **b** Venn diagram of upregulated and downregulated genes in the AC-hMSCs and nonAC-hMSCs at 24 h and 72 h. **c** VEGFA mRNA and **d** protein expression levels in AC-hMSCs and nonAC-hMSCs at 24 h and 72 h were measured by RT-qPCR and western blotting, respectively. **e** VEGFA secretion from hMSCs as determined by ELISA. Data are represented as mean ± SD (*n* = 3 per group). ^∗^*P* < 0.05

We first used flow cytometry to identify MSCs and found that hMSCs were positive for CD73, CD90, and CD105, whereas negative for CD11b, CD14, CD34, and CD45 (Additional file 1: Figure S1). Next, microarray analysis was performed in order to profile mRNAs in AC-hMSCs and nonAC-hMSCs at two different time points (Fig. 1a; Additional file 2: Table S1; Additional file 3: Table S2). Two thousand four hundred ninety-six mRNAs were downregulated (1663 genes at 24 h and 1820 genes at 72 h), and 2400 mRNAs were upregulated (1489 genes at 24 h and 1766 genes at 72 h) in nonAC-hMSCs when compared to those in AC-hMSCs (Fig. 1b). The mRNA expression profiles from nonAC-hMSCs at two different time points showed some overlap, with 987 downregulated mRNAs and 855 upregulated mRNAs shared between both samples (Fig. 1b). The 50 most downregulated and 50 most upregulated mRNAs in nonAC-hMSCs at both time points are shown as a heat map in Fig. 1a and are listed in Table 1. The expression of VEGFA in the nonAC-hMSCs was significantly higher than that in the AC-hMSCs at the two time points (Table 1). This increase in VEGFA mRNA in the nonAC-hMSCs was also verified by RT-qPCR. Western blot results showed that the VEGFA protein level was higher in nonAC-hMSCs than in AC-hMSCs (Fig. 1c, d; Additional file 4: Figure S2A). Moreover, the secretion of VEGFA, as determined by ELISA, was significantly higher in nonAC-hMSCs than in AC-hMSCs (Fig. 1e). Taken together, these results indicate that the nonadherent culture method elevates the expression of VEGFA in hMSCs and facilitates VEGFA secretion.

**Table 1:** Differential expression of genes in AC-hMSCs and nonAC-hMSCs at two different time points

Gene name	24 h			72 h		
	Fold change	*p* value	Regulation	Fold change	*p* value	Regulation
C2CD4B	111.8401912	0.000145643	Up	1340.34	0.000682199	Up
SSTR2	48.09021308	0.000555557	Up	307.6615	0.000223379	Up
SLC16A6	58.22005835	0.000188778	Up	244.5447	0.000546686	Up
FAM65C	19.69493511	2.3287E-08	Up	213.9348	3.43255E-05	Up
WDR86	29.35821269	2.03996E-05	Up	190.5289	6.26129E-05	Up
GJB2	33.73311298	2.37009E-05	Up	170.4077	0.000560036	Up
CXCR4	36.63486255	0.000412617	Up	156.6291	0.000381246	Up
MMP13	18.1969967	0.001865303	Up	119.5122	1.05064E-07	Up
BMP2	20.03644612	0.000193438	Up	117.9299	2.56801E-05	Up
C2CD4A	14.64162866	0.0061597	Up	85.52482	0.000625896	Up
CLEC2B	13.20455766	0.000159395	Up	78.42479	0.002468774	Up
TNFSF10	29.04491724	0.001267072	Up	57.67481	0.000386508	Up
PRSS35	11.75846586	0.000587899	Up	56.35998	0.000564307	Up
MEGF10	21.16086411	0.002528035	Up	55.27018	5.18695E-05	Up
DUSP4	13.21396805	9.34513E-05	Up	54.87385	0.001212616	Up
TSPAN11	27.83430153	0.000297067	Up	53.68215	9.89532E-07	Up
DRAXIN	13.45155294	0.000251701	Up	51.35024	0.000127201	Up
PDK4	96.98929796	5.63176E-05	Up	46.77971	2.99919E-05	Up
ANKH	10.78789239	0.004008663	Up	46.26112	2.57026E-05	Up
RGS16	39.28019753	0.000545809	Up	45.46778	7.36326E-07	Up
RANBP3L	9.229649839	5.07326E-06	Up	38.70846	7.23795E-05	Up
ITGA2	21.59037248	5.13189E-05	Up	38.41117	0.000872801	Up
ADGRE2	9.283517607	0.008575709	Up	34.87146	6.97014E-06	Up
MMP11	16.87338081	0.00285751	Up	34.62489	0.001476287	Up
KLRK1	11.95483946	0.000405053	Up	34.56032	0.002009842	Up
BEGAIN	33.51924802	6.25637E-07	Up	30.53104	0.000704421	Up
PPL	9.869982413	0.009031407	Up	25.65778	0.000467585	Up
ABCG1	30.41288703	0.000525295	Up	24.69536	4.16571E-07	Up
CITED1	18.69977736	0.000106336	Up	24.55424	0.000276694	Up
SYTL5	36.85560246	0.000548581	Up	23.16011	0.001025661	Up
RGS17	12.57031107	0.000155551	Up	22.45184	0.000241119	Up
KIAA1211L	16.30985794	1.57458E-05	Up	22.31267	3.31962E-05	Up
GLDN	38.48393676	0.000844764	Up	21.32752	0.000790957	Up
EXPH5	15.81689248	0.001356464	Up	20.98551	0.001995397	Up
PTGDR	14.69735545	0.002868926	Up	18.19271	0.000615557	Up
CCR1	19.99097768	7.95976E-05	Up	16.40048	0.002030008	Up
RASSF10	32.93415505	0.002178622	Up	16.05662	0.000741795	Up
FAM189A1	11.84572522	0.005621653	Up	15.56768	1.4842E-06	Up
COLEC12	23.05965011	0.000724925	Up	15.25867	0.00408674	Up
KLRC1	10.05484612	0.002881202	Up	15.20619	0.000154447	Up
VEGFA	9.752636526	4.13371E-06	Up	14.73218	0.000346357	Up
XKRX	16.61076595	0.000501039	Up	14.38702	0.000172706	Up
RASGRP3	2.106425558	0.028207257	Up	13.9441	7.03918E-05	Up
ENTPD3	9.912879304	0.001624712	Up	13.76377	0.000782473	Up
YPEL4	53.61892782	3.5446E-05	Up	13.63856	0.002333755	Up
SLC7A8	11.49043722	0.011671519	Up	13.22151	0.002134691	Up
CH25H	30.44303155	0.001762942	Up	12.42755	0.004566846	Up
CYFIP2	2.709902602	0.007297695	Up	10.76685	8.22518E-05	Up
C3AR1	8.089947094	0.000411377	Up	9.559041	0.000110391	Up
F2RL1	8.771762767	0.000415707	Up	9.21932	0.000385279	Up
CPA4	0.060443451	0.004894715	Down	0.005735	3.23423E-05	Down
CNN1	0.071987351	0.002896126	Down	0.005827	0.001199281	Down
MEST	0.041677656	0.002021602	Down	0.006979	1.73885E-05	Down
KRT34	0.011913749	0.00081183	Down	0.007354	5.15434E-05	Down
DKK1	0.004639044	0.000883335	Down	0.010773	4.20121E-05	Down
LMCD1	0.029802085	2.33205E-05	Down	0.012196	0.0006374	Down
KRTAP2-3	0.011067704	0.000539452	Down	0.015092	0.000257229	Down
FAM46B	0.033933237	4.34704E-06	Down	0.0174	0.00090771	Down
MYBL1	0.029145858	1.44931E-05	Down	0.017623	0.001442927	Down
NEIL3	0.017315518	0.000108138	Down	0.01963	0.00027453	Down
KRTAP1-5	0.029877632	0.001686929	Down	0.019696	0.000129879	Down
SPC25	0.044301408	0.001309748	Down	0.027418	0.001322887	Down
THBS1	0.055229076	0.000388028	Down	0.028426	0.002471628	Down
PTX3	0.010465082	0.001271904	Down	0.028936	3.57597E-05	Down
TSPAN18	0.053314954	0.000181383	Down	0.029322	0.007596185	Down
KRTAP2-2	0.048512804	1.88464E-05	Down	0.029763	0.00057951	Down
MGAM	0.034235468	0.00061814	Down	0.030484	0.001303756	Down
ANLN	0.05142361	0.001527223	Down	0.031571	0.001433314	Down
KIRREL3	0.057630141	0.002929558	Down	0.03209	0.000359608	Down
E2F8	0.018025232	1.75885E-05	Down	0.032301	0.000149989	Down
CEP55	0.0606109	0.000871839	Down	0.033875	0.000278821	Down
CTGF	0.048584673	0.000553306	Down	0.034772	0.000177803	Down
SKA1	0.04626685	1.04503E-05	Down	0.034872	0.000282226	Down
KRTAP1-1	0.029512423	2.68546E-06	Down	0.036294	0.001195564	Down
KRTAP1-4	0.034637793	8.67165E-05	Down	0.032924	0.000393189	Down
SHCBP1	0.074874368	0.007927709	Down	0.037302	4.80244E-05	Down
CDC45	0.048785792	0.001757321	Down	0.038191	0.002567892	Down
MKI67	0.061895471	0.001531656	Down	0.039185	0.006627038	Down
ERCC6L	0.031855904	1.35451E-06	Down	0.040946	0.001070963	Down
TNFRSF11B	0.035805553	0.004205691	Down	0.041701	0.000357158	Down
ZNF367	0.048382669	0.003338013	Down	0.042294	8.8958E-05	Down
DMD	0.038750757	1.1526E-05	Down	0.042472	0.001858382	Down
CDCA8	0.038925122	0.000236484	Down	0.043372	0.000204384	Down
PADI1	0.041441366	0.000851724	Down	0.044515	7.47813E-06	Down
HIST2H3C	0.045817546	0.00054878	Down	0.045124	0.000144333	Down
TTK	0.061307423	7.72508E-05	Down	0.045391	0.000936671	Down
GABBR2	0.045546263	0.001708625	Down	0.045807	9.62305E-05	Down
FAM111B	0.027357567	4.93311E-06	Down	0.046582	0.000558007	Down
CCIN	0.051329319	0.003246805	Down	0.048819	5.93123E-05	Down
KIF15	0.066141532	0.000117669	Down	0.04886	0.001136135	Down
NCKAP5	0.066816867	0.000303257	Down	0.049423	0.001324705	Down
NTF3	0.042448723	0.001215131	Down	0.050437	0.005324032	Down
UBE2C	0.065284894	4.29692E-06	Down	0.052099	0.002768879	Down
KRT33B	0.064358986	0.00039797	Down	0.052188	0.000124335	Down
MCM10	0.046540673	0.000257847	Down	0.054035	0.003217037	Down
LMOD1	0.054276425	1.14094E-06	Down	0.055313	0.000663673	Down
LTF	0.068482765	1.6193E-05	Down	0.057596	0.000575741	Down
HIST1H3G	0.035683104	0.000992525	Down	0.058962	0.000146762	Down
CCDC85A	0.066799698	9.312090059	Down	0.059079	0.009067666	Down
NCAPG	0.073153137	6.164797229	Down	0.060212	0.037036192	Down

### Decreased levels of VEGFA produced by VEGFA knockdown nonAC-hMSCs inhibit the angiogenesis of HUVECs

**Fig. 2:**
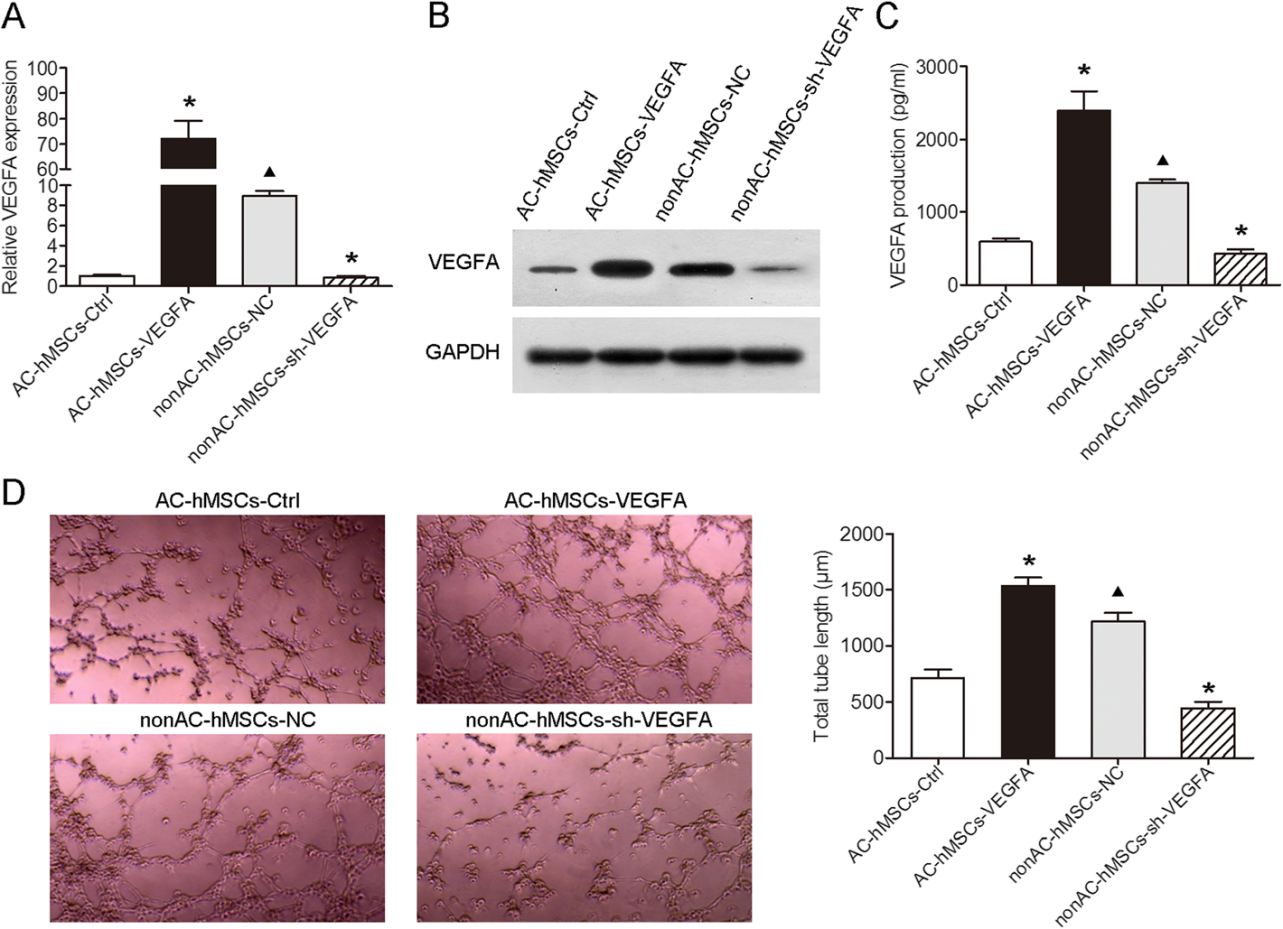
Decreased levels of VEGFA produced by VEGFA knockdown nonAC-hMSCs inhibits the angiogenesis of HUVECs. AC-hMSCs were infected with VEGFA or negative control (NC) lentiviruses and nonAC-hMSCs were infected with shVEGFA or the control lentiviruses. **a** RT-qPCR and **b** western blotting were performed to detect mRNA and protein levels of VEGFA, respectively. **c** ELISA analysis was performed to determine the levels of secreted VEGF. **d** Capillary-like structure formation of HUVECs cultured in CM from hMSCs with different genotypes was evaluated by tube formation assay. Data are represented as mean ± SD (*n* = 3 per group). ^∗^*P* < 0.05 compared to AC-hMSC-Ctrl or nonAC-hMSC-NC group. ^▲^*P* < 0.05 compared to AC-hMSC-Ctrl group

A lentivirus-based system was used to assess whether the expression of VEGFA in hMSCs plays an important role in HUVEC angiogenesis. VEGFA was stably overexpressed in AC-hMSCs (AC-VEGFA-hMSCs) and depleted in nonAC-hMSCs (nonAC-shVEGFA-hMSCs) (Fig. 2a, b; Additional file 4: Figure S2B). ELISA analysis revealed that forced expression of VEGFA induced VEGFA production, whereas knockdown of VEGFA led to the converse (Fig. 2c). We performed a tube formation assay to detect angiogenesis of HUVECs and found that HUVECs cultured in CM from AC-VEGFA-hMSCs and nonAC-hMSCs formed well-developed networks of capillary-like tubes. In contrast, HUVECs cultured in CM from nonAC-shVEGFA-hMSCs and AC-hMSCs displayed very few capillary-like structures (Fig. 2d). Collectively, these data suggest that silencing and therefore limiting secretion of VEGFA in nonAC-hMSCs hindered tube formation of HUVECs, whereas excess VEGFA secretion in AC-hMSCs promoted HUVEC tube formation.

### VEGFA production by VEGFA-overexpressing AC-hMSCs increases angiogenesis and reduces infarction size after MI

**Fig. 3:**
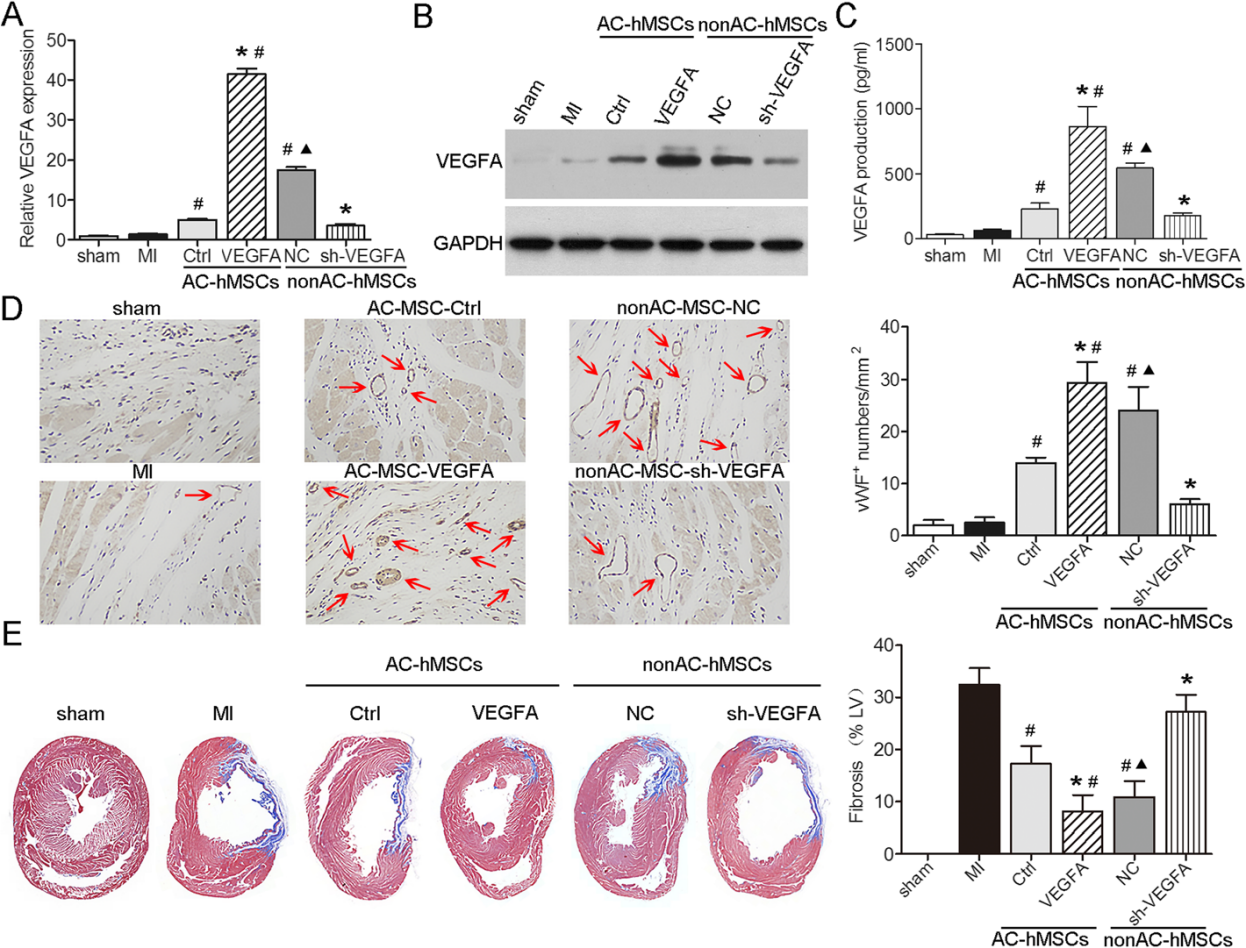
VEGFA production by VEGFA-overexpressing AC-hMSCs increases angiogenesis and reduces infarction size after MI. **a** RT-qPCR, **b** western blotting, and **c** ELISA were used to assess VEGFA expression and secretion in the heart tissues obtained from LADCA-ligated rats 48 h after transplantation with AC-VEGFA-hMSCs, nonAC-sh-VEGFA-hMSCs, or their corresponding control hMSCs. **d** Immunohistochemistry analysis for vWF expression in the heart tissues obtained from LADCA-ligated rats 28 days after transplantation with AC-VEGFA-hMSCs, nonAC-sh-VEGFA-h MSCs, or their corresponding control hMSCs. Red arrow indicates the small vessels. **e** Representative images and quantification of fibrosis area. Data are represented as mean ± SD (*n* = 8 per group). ^∗^*P* < 0.05 compared to AC-hMSC-Ctrl or nonAC-hMSC-NC group. ^#^*P* < 0.05 compared to MI group. ^▲^*P* < 0.05 compared to AC-hMSC-Ctrl group

To explore the functional significance of VEGFA in MSC-mediated angiogenesis in a rat MI model, we transplanted AC-VEGFA-hMSCs, nonAC-shVEGFA-hMSCs, or their corresponding control cells into the ischemic LV wall border zone and analyzed VEGFA expression and secretion after cell transplantation. Compared to the corresponding control groups, AC-VEGFA-hMSC-transplanted group and nonAC-hMSC-transplanted group had significantly higher levels of VEGFA expression and secretion, whereas nonAC-shVEGFA-hMSC-transplanted group and AC-hMSC-transplanted group exhibited significantly lower levels of VEGFA expression and secretion (Fig. 3a–c; Additional file 4: Figure S2C). Furthermore, we evaluated angiogenesis in the infarcted heart after hMSC transplantation using immunohistochemistry and found that the number of vWF-positive vessels significantly increased in AC-VEGFA-hMSC- and nonAC-hMSC-treated groups, while they were markedly decreased in nonAC-shVEGFA-hMSC- and AC-hMSC-treated groups (Fig. 3d). Additionally, we used Masson’s trichrome staining to detect the extent of fibrosis after hMSC transplantation. The results showed that the infarct size was significantly smaller in the AC-VEGFA-hMSC-injected hearts and nonAC-hMSC-injected hearts than in the control AC-hMSC-injected hearts, whereas it was significantly larger in the nonAC-shVEGFA-hMSC-injected hearts and AC-hMSC-injected hearts than in the control nonAC-hMSC-injected hearts (Fig. 3e). Therefore, our data demonstrate that implantation of AC-VEGFA-hMSCs promotes angiogenesis and decreases infarction size by stimulating VEGFA production in the rat MI model.

### VEGFA secretion by VEGFA-overexpressing AC-hMSCs improves cardiac functions in a rat MI model

**Fig. 4:**
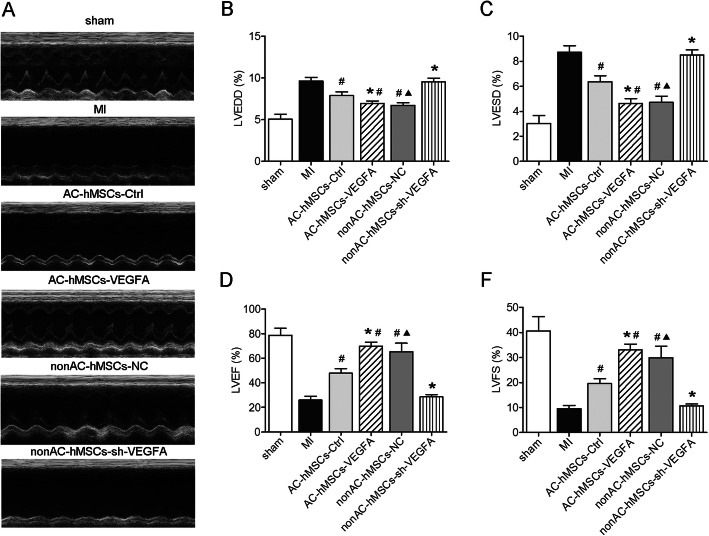
VEGFA secretion by VEGFA-overexpressing AC-hMSCs improves cardiac functions in a rat MI model. **a** Cardiac function was determined by echocardiography 28 days after MI induction and hMSC transplantation. **b** Quantification of LVEDD, **c** LVESD, **d** LVEF, and **e** LVFS. Data are represented as mean ± SD (*n* = 8 per group). ^∗^*P* < 0.05 compared to AC-hMSC-Ctrl or nonAC-hMSC-NC group. ^#^*P* < 0.05 compared to MI group. ^▲^*P* < 0.05 compared to AC-hMSC-Ctrl group

Echocardiography was performed 4 weeks after hMSC implantation in a rat MI model to assess the therapeutic effects of MSCs on cardiac function (Fig. 4a). LVEDD and LVESD were significantly lower in the AC-VEGFA-hMSC and nonAC-hMSC groups and significantly higher in the nonAC-shVEGFA-hMSC and AC-hMSC groups compared to the corresponding control groups (Fig. 4b, c). Furthermore, the AC-VEGFA-hMSC and nonAC-hMSC groups exhibited significantly increased LVEF and LVFS, whereas the nonAC-shVEGFA-hMSC and AC-hMSC groups displayed significantly decreased LVEF and LVFS (Fig. 4d, e). Collectively, these findings demonstrate that implantation of VEGFA-overexpressing AC-hMSCs could decrease myocardial remodeling.

### VEGFA is a direct target of miR-519d

**Fig. 5:**
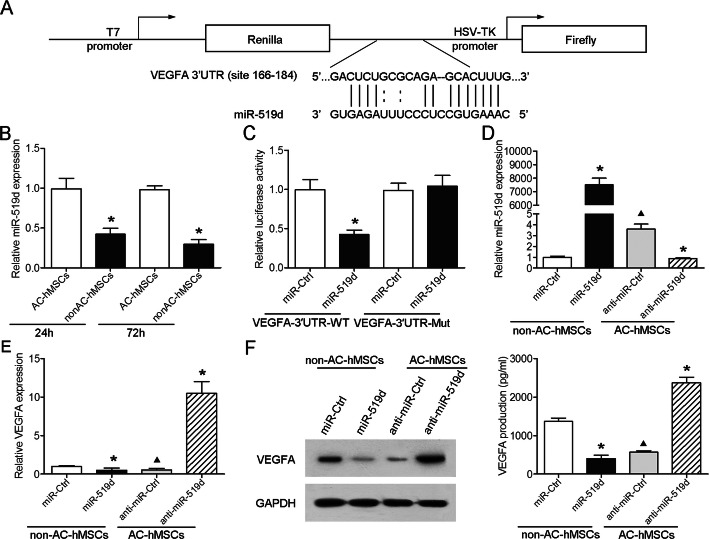
VEGFA is a direct target of miR-519d. **a** The predicted binding site between VEGFA and miR-519d. **b** RT-qPCR analysis of miR-519d expression in AC-hMSCs and nonAC-hMSCs at 24 h and 72 h. **c** Luciferase activity in HEK293T cells co-transfected with miR-519d or control mimics and VEGFA-3′ UTR-WT or VEGFA-3′ UTR-Mut plasmids. **d–f** VEGFA expression and secretion in nonAC-hMSCs transfected with miR-519d or control mimics, and AC-hMSCs transfected with miR-519d or control inhibitors. Data are represented as mean ± SD (*n* = 3 per group). ^∗^*P* < 0.05 compared to miR-Ctrl or anti-miR-Ctrl group. *P* < 0.05 compared to miR-Ctrl group

To elucidate the molecular mechanism of VEGFA in the regulation of angiogenesis, we first employed the online bioinformatics (TargetScan and microrna.org) to predict the potential microRNA (miRNA) binding sites in VEGFA. miR-519d was found to be a putative miRNA for the regulation of VEGFA (Fig. 5a). We then probed for miR-519d expression in AC-hMSCs and nonAC-hMSCs at two different time points using RT-qPCR and found that miR-519d expression was significantly lower in nonAC-hMSCs compared to AC-hMSCs (Fig. 5b). As shown in Fig. 1c, d, the expression of VEGFA was significantly higher in nonAC-hMSCs than in AC-hMSCs. This negative correlation between VEGFA and miR-519d expression indicates that miR-519d may be involved in the regulation of VEGFA.

To validate a direct interaction between VEGFA and miR-519d, a luciferase reporter plasmid containing wild-type or mutant VEGFA 3′ UTR was constructed and transfected into HEK293T cells, along with miR-519d or control mimics. Results showed that miR-519d led to a significant reduction in the luciferase activity produced by the construct containing the VEGFA putative miR-519d binding site, whereas miR-519d had no significant effect on the construct containing the mutant VEGFA 3′ UTR (Fig. 5c). Furthermore, RT-qPCR, western blot, and ELISA showed that overexpression of miR-519d in nonAC-hMSCs significantly decreased the expression and secretion of VEGFA, and knockdown of miR-519d in AC-hMSCs enhanced VEGFA expression and secretion (Fig. 5d–f; Additional file 4: Figure S2D). Taken together, these results indicate that miR-519d exerts suppressive effects on VEGFA expression by binding the VEGFA 3′ UTR.

### The secretion of VEGFA in VEGFA-overexpressing nonAC-hMSCs attenuates miR-519d-mediated inhibition of tube formation of HUVECs

**Fig. 6:**
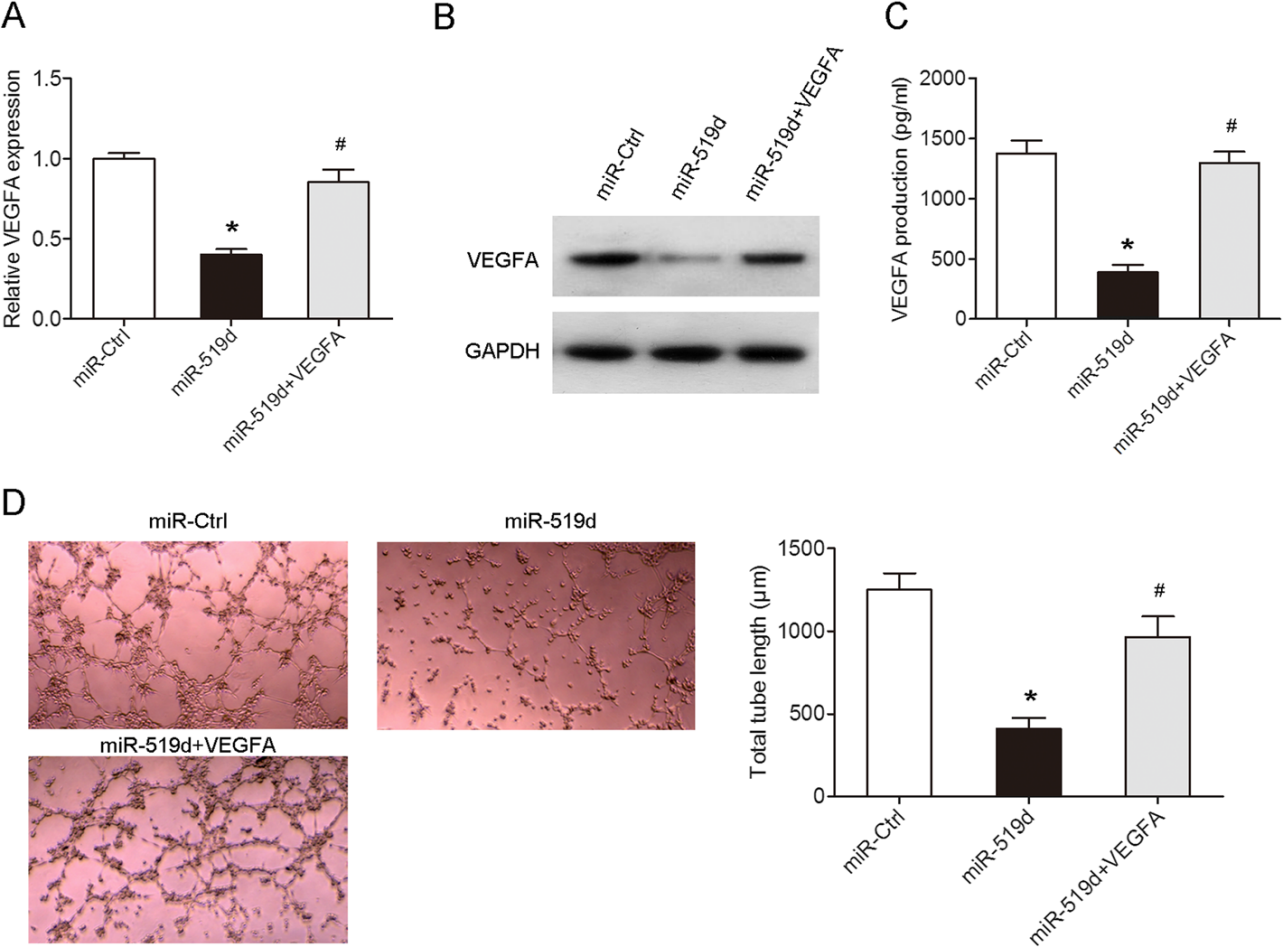
The secretion of VEGFA in VEGFA-overexpressing nonAC-hMSCs attenuates miR-519d-mediated inhibition of tube formation of HUVECs. **a** The expression and secretion of VEGFA in the nonAC-hMSCs co-transfected with miR-519d mimics and VEGFA plasmid were measured using RT-qPCR, **b** western blotting, and **c** ELISA, respectively. **d** Tube formation assay was performed to detect the capillary-like tubules formation of HUVECs cultured in the presence of hMSC-conditioned medium. Data are represented as mean ± SD (*n* = 3 per group). ^∗^*P* < 0.05 compared to miR-Ctrl group. ^#^*P* < 0.05 compared to miR-519d group

We performed a rescue experiment to determine whether miR-519d regulates cell angiogenesis via targeting VEGFA. nonAC-hMSCs were co-transfected with miR-519d mimics and VEGFA plasmids for 24 h. RT-qPCR and western blot analyses indicated that ectopic expression of VEGFA attenuated miR-519d-mediated suppression of VEGFA expression (Fig. 6a, b; Additional file 4: Figure S2E) and secretion (Fig. 6c). Furthermore, while miR-519d-overexpressing decreased tube formation of HUVECs, the addition of VEGFA derived from VEGFA-overexpressing nonAC-hMSCs reversed this effect (Fig. 6d). Collectively, these data suggest that miR-519d exerts its biological effects by directly targeting VEGFA.

## Discussion

A growing body of evidence suggests that the beneficial therapeutic effects of MSCs are the result of paracrine mechanisms [26, 27]. Various growth factors, cytokines, and angiogenic factors released from MSCs play critical roles in improving the function of infracted hearts [28, 29]. VEGFA belongs to the VEGF family and is a well-known pro-angiogenic factor. VEGFA has been reported to stimulate endothelial cell proliferation, migration, and tube formation, thereby promoting angiogenesis [30]. Recent studies indicated that VEGFA released from different types of stem cells, including MSCs, have the ability to treat various human diseases, such as myocardial ischemia, by regulation of angiogenesis [31–33]. Markel et al. [31] reported that VEGFA knockdown in MSCs impaired stem cell-mediated myocardial function. Cho et al. [32] reported that VEGFA-secreting human umbilical cord blood-derived MSCs decreased infarct size and improved cardiac function via enhancing angiogenesis in infarct myocardium. Kim et al. [23] also reported that the transplantation of hypoxia inducible VEGFA-expressing MSCs promoted ischemia-responsive VEGFA production, resulting in a significant decrease in apoptosis and a significant increase in micro-vessel formation after MI. In our study, VEGFA was upregulated in nonAC-hMSCs (Fig. 1). The high levels of VEGFA secreted by AC-VEGFA-hMSCs facilitated HUVEC angiogenesis, while the low expression levels of VEGFA produced by nonAC-shVEGFA-hMSCs suppressed this process (Fig. 2). In a rat MI model, administration of AC-VEGFA-hMSCs significantly increased VEGFA production, leading to improvement in myocardial function, enhancement in vessel density, and reduction in infarct size, whereas reversed results were observed in nonAC-shVEGFA-hMSC-transplanted hearts (Figs. 3 and 4). Recently, it has been reported that VEGFA secretion protects cardiomyocytes against ischemia [33]. Additionally, VEGF is known to modulate macrophage recruitment and macrophage phenotype by promoting M1 ≥ M2 transpolarization [34, 35]. In skin cancer, VEGFA promotes macrophage recruitment to the tumor and thereby facilitates tumor-associated macrophage development [34]. In chronic kidney disease, ELP-VEGF therapy distinctly shifted renal macrophage phenotype from proinflammatory M1 to VEGF expressing M2, restoring VEGF signaling and sustaining improvement of renal function and microvascular integrity [35]. However, whether VEGFA improves cardiac function by affecting cardiomyocytes or regulating macrophage recruitment and macrophage phenotype needs further investigation.

A number of studies have indicated that VEGFA can be directly regulated by miRNAs [36, 37]. miR-299-3p inhibited cell proliferation and motility and induced apoptosis in renal cell carcinoma by directly targeting VEGFA [36]. miR-199a-5p impaired both the survival and angiogenic capacity of MSCs by directly regulating VEGFA expression [37]. In our study, miR-519d directly affected VEGFA expression (Fig. 5). miR-519d is one member of the chromosome 19 miRNA cluster, which is the largest human miRNA cluster containing 46 pre-miRNAs [38]. There is growing evidence showing that miR-519d is involved in a range of biological processes, including cell proliferation, apoptosis, differentiation, migration, and angiogenesis [39–42]. miR-519d overexpression facilitated the proliferation, migration, invasion, and adhesion of melanoma cells in vitro and accelerated lung metastatic capability in vivo [39]. Expression of miR-519d promoted hepatocellular carcinoma cell proliferation and invasion and inhibited apoptosis [40]. On the other hand, overexpression of miR-519d impaired colorectal cancer cell viability, migration, and invasion and induced G0/G1 phase arrest and apoptosis by downregulating TROAP expression [41]. Additionally, miR-519d expression attenuated endothelial cell growth, migration, and tube formation [42]. In this study, increased VEGFA production in nonAC-hMSCs reversed the suppressive effect of miR-519d on HUVEC tube formation (Fig. 6).

How does the nonadherent culture method regulate the expression of miR-519d? Our previous research showed that the nonadherent culture method decreased Sca-1 expression [14]. Our microarray analysis also demonstrated that the nonadherent culture method upregulated CXCR4 expression. Based on these data, we hypothesized that the nonadherent culture method might regulate the expression of miR-519d by regulating the adhesion molecules of MSCs. Further investigation is needed to verify this hypothesis.

## Conclusions

In summary, we determined that the nonadherent culture of MSCs increased the secretion of VEGFA, directly opposing the effects of miR-519d (Additional file 5: Figure S3). VEGFA promoted the tube formation capabilities of HUVECs in vitro, decreased myocardial remodeling and infarct size, and increased angiogenesis in a rat MI model. These findings provide a novel experimental protocol to optimize MSC-based therapies for the treatment of MI.

## Supplementary information

**Additional file 1 : Figure S1.** Characterization of isolated hMSCs. Flow cytometry showing the percentage of CD73, CD90, CD105, CD11b, CD14, CD34 and CD45 hMSCs.

**Additional file 2 : Table S1.** Differential expression of genes in AC-hMSCs and nonAC-hMSCs at 24 h.

**Additional file 3 : Table S2.** Differential expression of genes in AC-hMSCs and nonAC-hMSCs at 72 h.

**Additional file 4 : Figure S2.** Relative VEGFA protein expression in the different groups. Data are represented as mean ± SD (*n* = 3 per group). ^∗^*P* < 0.05 compared to AC-hMSCs, AC-hMSCs-Ctrl, nonAC-hMSCs-NC, miR-Ctrl or anti-miR-Ctrl group. ^#^*P* < 0.05 compared to miR-519d group. *P* < 0.05 compared to AC-hMSCs-Ctrl or miR-Ctrl group.

**Additional file 5 : Figure S3.** The schematic diagram shows how VEGFA expression is regulated by miR-519d-3p after changes of adhesion.

## Data Availability

All data generated and/or analyzed during the current study are included in this manuscript.

## Abbreviations

MSCs: Mesenchymal stem cells
MI: Myocardial infarction
hMSCs: Human MSCs
AC-hMSCs: hMSCs grown as adherent cultures
nonAC-hMSCs: hMSCs grown as nonadherent cultures on ultra-low-adherent plates
AC-VEGFA-hMSCs: VEGFA-overexpressing AC-MSCs
nonAC-shVEGFA-hMSCs: VEGFA-knockdown nonAC-hMSCs
RT-qPCR: Real-time quantitative polymerase chain reaction
ELISA: Enzyme-linked immunosorbent assays
HUVECs: Human umbilical vein endothelial cells
Sca-1: Stem cell antigen 1
LADCA: Left anterior descending coronary artery
LV: Left ventricle
LVEDD: LV end-diastolic dimensions
LVESD: LV end-systolic dimensions
