# Supplementary figures and images for: Nonadherent culture method promotes MSC-mediated vascularization in myocardial infarction via miR-519d/VEGFA pathway
Source: Stem Cell Res Ther. 2020 Jul 2;11:266. doi: 10.1186/s13287-020-01780-x (PMC7330937; doi:10.1186/s13287-020-01780-x)

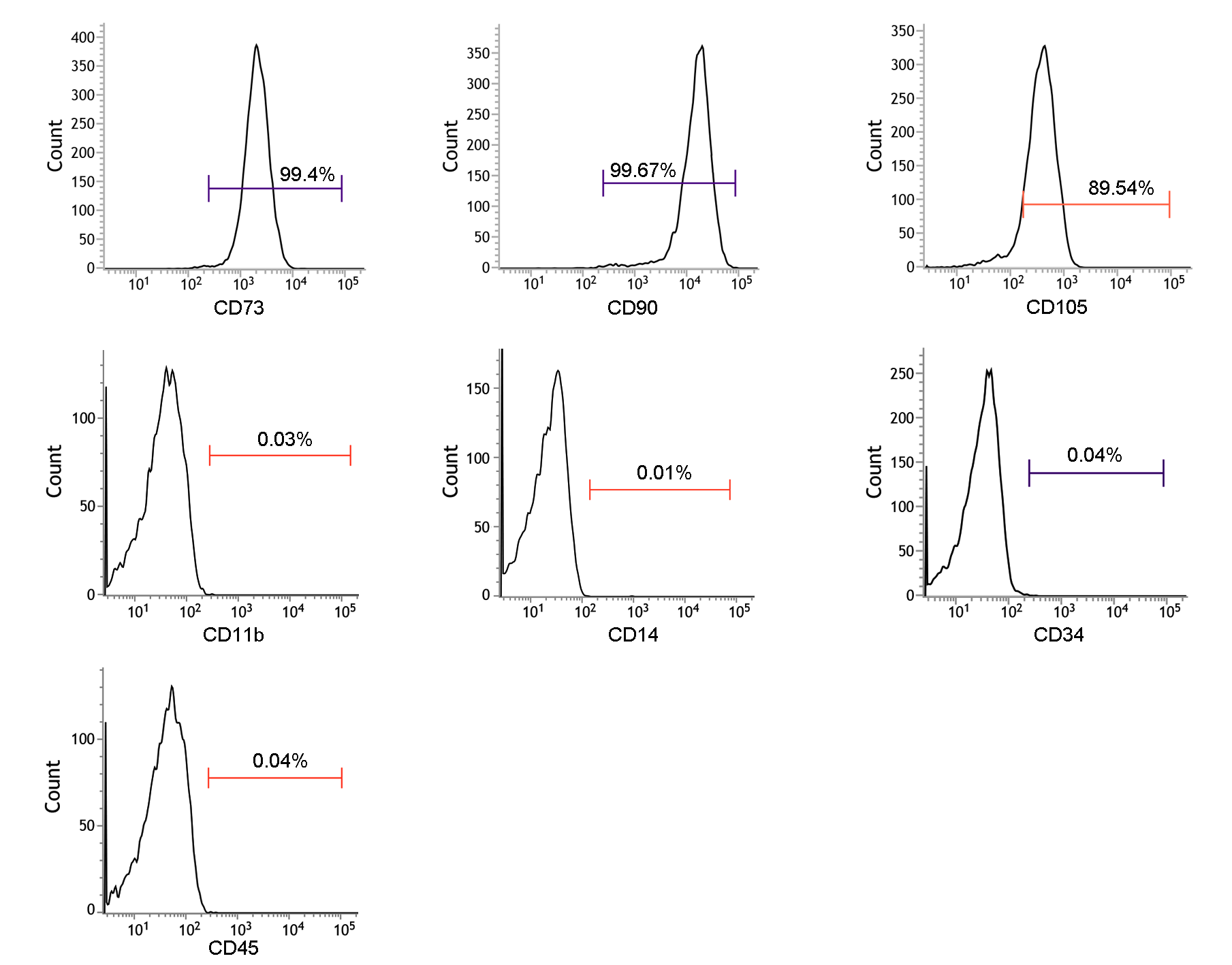

Supplement: Supplementary file 1 — Additional file 1 : Figure S1. Characterization of isolated hMSCs. Flow cytometry showing the percentage of CD73, CD90, CD105, CD11b, CD14, CD34 and CD45 hMSCs. [file 13287_2020_1780_MOESM1_ESM.tif]

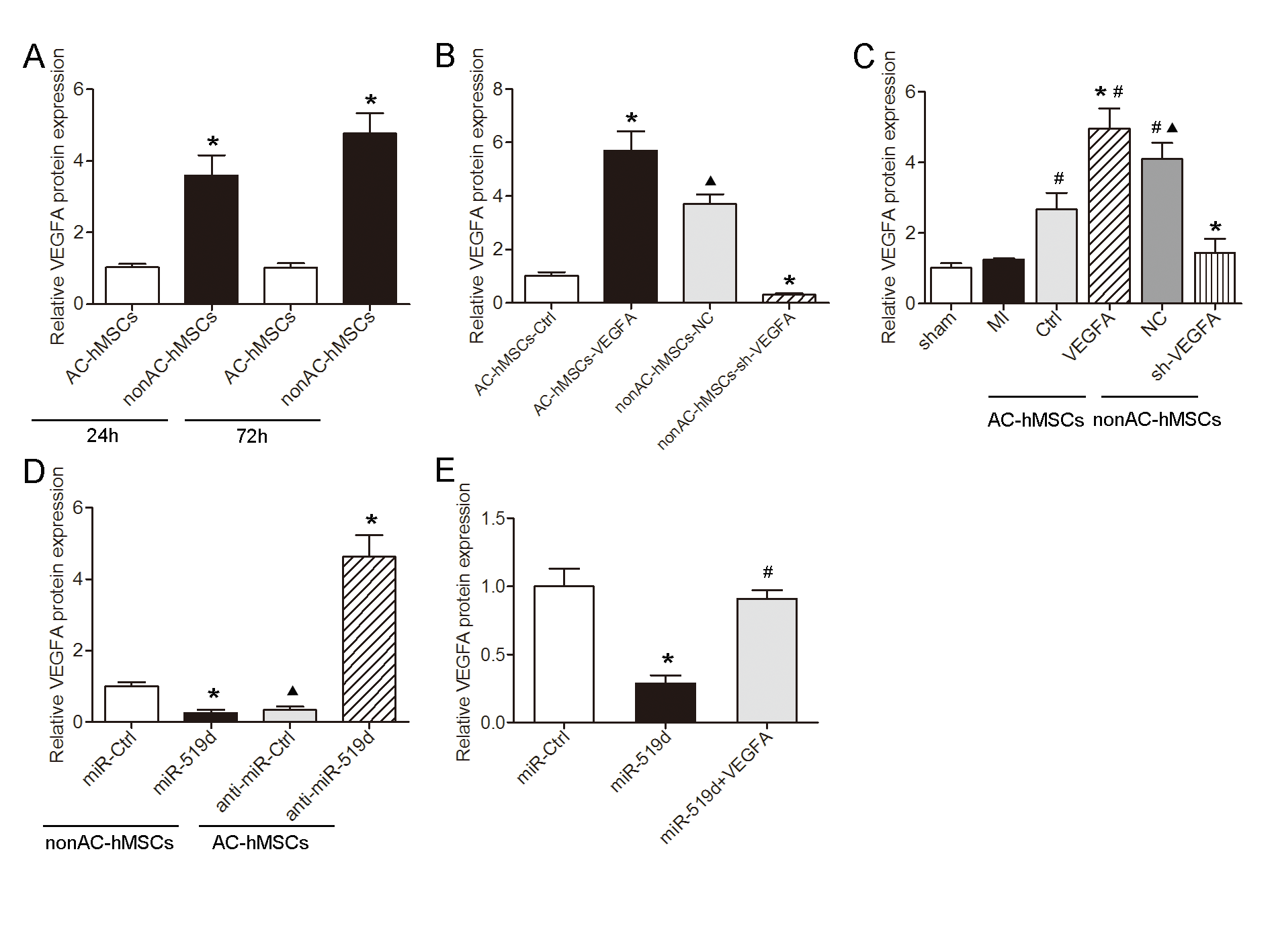

Supplement: Supplementary file 4 — Additional file 4 : Figure S2. Relative VEGFA protein expression in the different groups. Data are represented as mean ± SD (n = 3 per group). ∗P < 0.05 compared to AC-hMSCs, AC-hMSCs-Ctrl, nonAC-hMSCs-NC, miR-Ctrl or anti-miR-Ctrl group. #P < 0.05 compared to miR-519d group. P < 0.05 compared to AC-hMSCs-Ctrl or miR-Ctrl group. [file 13287_2020_1780_MOESM4_ESM.tif]

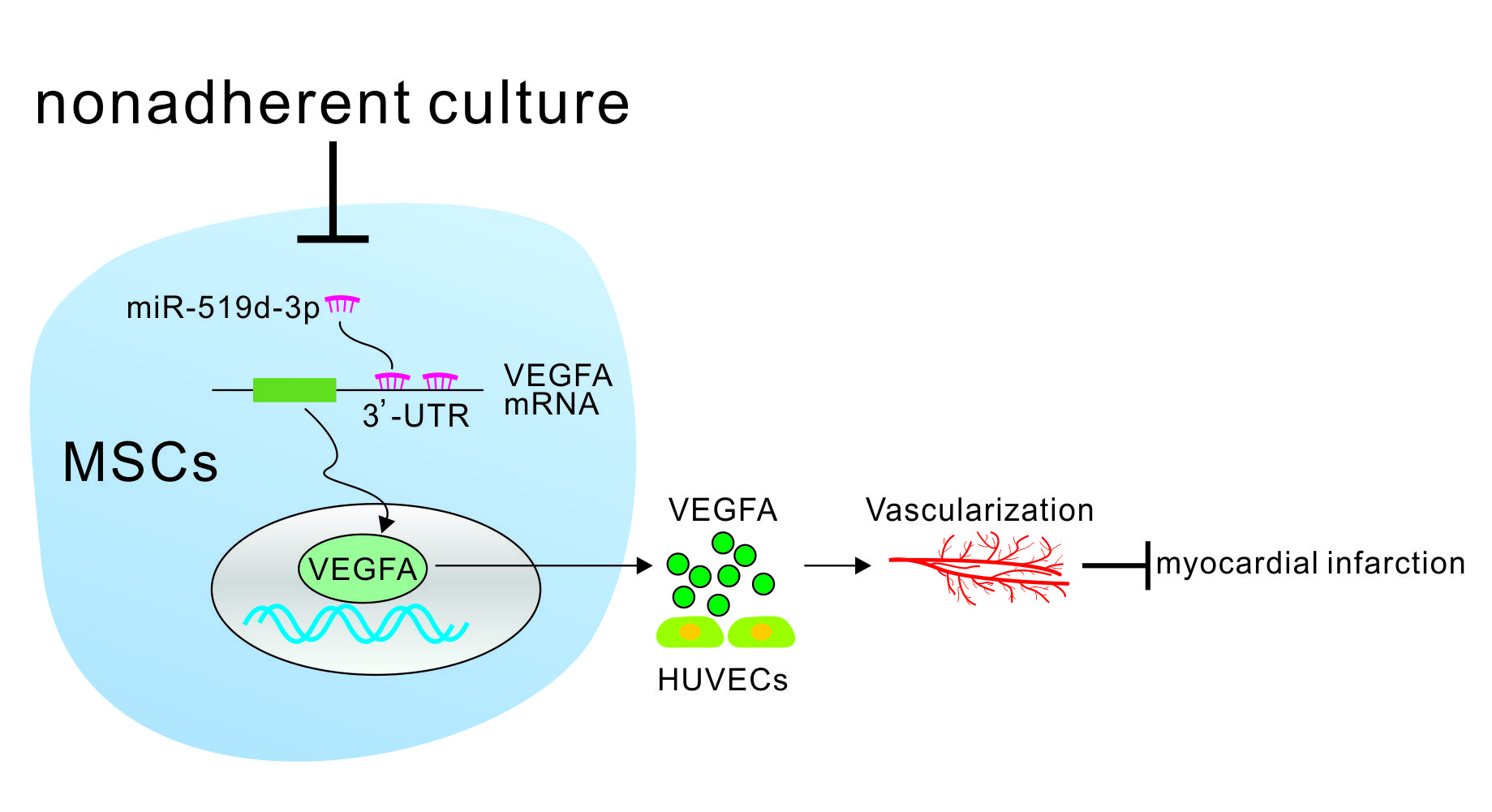

Supplement: Supplementary file 5 — Additional file 5 : Figure S3. The schematic diagram shows how VEGFA expression is regulated by miR-519d-3p after changes of adhesion. [file 13287_2020_1780_MOESM5_ESM.tif]
